# Intravenous ferric carboxymaltose for anaemia in pregnancy

**DOI:** 10.1186/1471-2393-14-115

**Published:** 2014-03-25

**Authors:** Bernd Froessler, Joshua Collingwood, Nicolette A Hodyl, Gustaaf Dekker

**Affiliations:** 1 Department of Anaesthesia, Lyell McEwin Hospital, Haydown Road, Elizabeth Vale 5112, South Australia, Australia; 2 Discipline of Acute Care Medicine, University of Adelaide, Adelaide, South Australia, Australia; 3 Ballarat Base Hospital, Drummond Street, North Ballarat, VIC, Australia; 4 NHMRC Peter Doherty Research Fellow, Robinson Institute, School of Paediatrics and Reproductive Health, University of Adelaide, Adelaide, South Australia, Australia; 5 Department of Obstetrics and Gynaecology, Lyell McEwin Hospital, Elizabeth Vale, South Australia, Australia; 6 Robinson Institute, Lyell McEwin Hospital, Elizabeth Vale, South Australia, Australia

**Keywords:** Pregnancy, Iron deficiency, Peri-partum anaemia, Intravenous ferric carboxymaltose, Red blood cell transfusion

## Abstract

**Background:**

Iron deficiency is a common nutritional deficiency amongst women of childbearing age. Peri-partum iron deficiency anaemia (IDA) is associated with significant maternal, fetal and infant morbidity. Current options for treatment are limited: these include oral iron supplementation, which can be ineffective and poorly tolerated, and red blood cell transfusions, which carry an inherent risk and should be avoided. Ferric carboxymaltose is a new treatment option that may be better tolerated.

The study was designed to assess the safety and efficacy of iron deficiency anaemia (IDA) correction with intravenous ferric carboxymaltose in pregnant women with mild, moderate and severe anaemia in the second and third trimester.

**Methods:**

Prospective observational study; 65 anaemic pregnant women received ferric carboxymaltose up to 15 mg/kg between 24 and 40 weeks of pregnancy (median 35 weeks gestational age, SD 3.6). Treatment effectiveness was assessed by repeat haemoglobin (Hb) measurements and patient report of well-being in the postpartum period. Safety was assessed by analysis of adverse drug reactions and fetal heart rate monitoring during the infusion.

**Results:**

Intravenous ferric carboxymaltose infusion significantly increased Hb values (p < 0.01) above baseline levels in all women. Increased Hb values were observed at 3 and 6 weeks post infusion and up to 8 weeks post-infusion. Ferritin values increased significantly after the infusion. Only 4 women had repeat ferritin values post-partum which remained above baseline levels. Fetal heart rate monitoring did not indicate a drug related negative impact on the fetus. Of the 29 (44.6%) women interviewed, 19 (65.5%) women reported an improvement in their well-being and 9 (31%) felt no different after the infusion. None of the women felt worse. No serious adverse effects were found and minor side effects occurred in 13 (20%) patients.

**Conclusions:**

Our prospective data is consistent with existing observational reports of the safe and effective use of ferric carboxymaltose in the treatment of iron deficiency anaemia in pregnancy.

## Background

Iron deficiency is recognized as a common nutritional deficiency amongst women of childbearing age in both the developed and developing world [1]. Peri-partum iron deficiency anaemia (IDA) is associated with significant maternal, fetal and infant morbidity. Poor outcomes for the fetus and infant include: preterm birth, fetal growth restriction, intrauterine fetal death, low Apgar scores and infection [2]. Women with iron deficiency are also at risk of adverse effects requiring medical interventions such as red blood transfusion [3], cardiovascular problems, reduced physical and cognitive performance, reduced immune function, tiredness and increased depressive episodes [4]. Peri-partum maternal iron deficiency has also been associated with childhood developmental problems [5] and negative mother-infant interactions such as an increase in negative statements and decreased responsiveness [6]. Progression from iron deficiency to iron deficiency anaemia (IDA) in pregnancy is common, due to the increased demand for iron during pregnancy, required to support maternal haemoglobin mass expansion, as well as the growing fetus and placenta [7]. This is further aggravated by blood loss associated with delivery. Deliveries by both caesarean section and vaginal deliveries that require instrumentation/intervention represent an even greater risk [4] increasing a woman’s vulnerability for peri-partum blood transfusion [3], chronic iron deficiency anaemia and iron store depletion, all compromising maternal well-being. However, this recognition has not resulted in a universal approach of iron supplementation [8].

Iron deficiency is potentially both preventable and treatable. Effective management strategies that allow women to replenish iron stores, both antenatal or during labour, restore haemoglobin values and are likely to enhance the health of the mother and infant [9]. For many decades the mainstay treatment of IDA has been oral iron and red blood cell (RBC) transfusions. However, oral iron supplementation can lead to significant side effects resulting in non-compliance in many patients [10] and the risks for RBC transfusion are well described and should be avoided whenever possible [11]. Intravenous iron formulations offer an alternative approach in the presence of moderate or severe anaemia, intolerance of or non-adherence to oral iron and malabsorption states [12]. Intravenous iron is less commonly used as fear of anaphylaxis with iron dextran formulations, and long infusion time with iron polymaltose, have led to reluctance amongst clinicians [13]. The development of dextran free parenteral iron formulations with an improved safety profile, and a more rapid delivery time suggests that intravenous iron should be considered as a mainstay treatment for moderate to severe IDA [14].

Iron Sucrose and Ferric Carboxymaltose are dextran free intravenous iron alternatives. When compared to oral iron in pregnancy iron sucrose is superior with respect to the rate of both haemoglobin increase and iron store replenishment, combined with a good safety profile [12,15,16]. Serious adverse effects are rare with iron sucrose, however minor side effects occur in up to 18% of patients which may in part be attributed to its non-physiological physical properties (high pH and high osmolarity). Ferric carboxymaltose is a newer dextran-free iron formulation with a near neutral pH, physiological osmolarity and increased bioavailability which allows for single dose, short 15 minute infusion time and higher dosing (up to 1000 mg) [17]. These properties make ferric carboxymaltose an attractive alternative to iron sucrose in terms of risk profile, efficacy, patient comfort and convenience, staff and institutional resource utilization.

To date, there are few clinical studies using ferric carboxymaltose in pregnant women. The primary aim of this study was to assess the use of intravenous ferric carboxymaltose in the correction of iron deficiency anaemia in pregnant women. The secondary aims were to determine the extent and severity of adverse effects of ferric carboxymaltose, and to evaluate the perceived quality of life of women in the post-partum period.

## Methods

After approval by the Queen Elizabeth Hospital, Lyell McEwin Hospital & Modbury Hospital Human Research and Ethics committee (Reference number 2011160) this prospective study was performed between July 2011 and September 2012. Informed consent was waived by the ethics committee for data collection, as ferric carboxymaltose was being used as our routine clinical treatment modality in this setting.

Pregnant women with documented IDA, defined as Hb < 115 g/dl, who consecutively presented as outpatients in the Women’s Assessment Unit at the Lyell McEwen Hospital (Elizabeth Vale South Australia) to receive ferric carboxymaltose infusions were recruited to this study. A total of 65 women were included. Due to the limited availability of safety data for its use in pregnancy, we adopted a longer infusion protocol (30 min) than recommended by the manufacturer (15 min). Maternal blood pressure was taken every five minutes during infusion and foetal heart rate was assessed before and after infusion. According to routine antenatal care blood samples were collected to measure haemoglobin, and in some cases ferritin levels, prior to infusion and then again, where clinically indicated, at up to three post-infusion visits (at approximately 3, 6 and 8 weeks). Haemoglobin and ferritin concentrations were determined in the hospital laboratory using Sodium lauryl sulphate (SLS) method for Hb analysis (Sysmex XE2100 analyser) and direct chemiluminometric sandwich immunoassay (Siemens ADVIA Centaur XP) for ferritin analysis. Women were observed for one hour post infusion, before being discharged home. Medical and pathology data were collated from case notes and electronic laboratory reports, as well as transfusion data linkage reports. A telephone interview was conducted after all 65 patients delivered to evaluate well-being after the infusion. Patients were asked to place themselves into 1 of 4 allocated categories (worse, no different, better or much better), which reflected degrees of perceived change in symptomatology since infusion.

The data were analysed using Graph Pad Prism 5, using p values of ≤0.05 to indicate significance. Available pre-infusion, post-infusion and post-partum haemoglobin, ferritin and transferrin saturation levels were compared using one-way ANOVA tests. Tukey’s multiple comparison tests were used to assess changes in levels across the time points measured with Dunn’s multiple comparison test used for post hoc analysis when necessary.

## Results

The characteristics of the women receiving ferric carboxymaltose for iron deficiency anaemia are outlined in Table 1. A total of 65 women received a ferric carboxymaltose infusion for antenatal iron deficiency anaemia, with pre-infusion haemoglobin data available for all 65 women. Following infusion, haemoglobin values were repeated by the obstetric team as required and data were available for 88% of women: 31 women (48%) at visit 1 (3 weeks post infusion), 26 women (40%) at visit 2 (6 weeks post infusion) and a total of 20 (31%) women had a blood test at visit 3 (8 weeks post infusion; post-partum). All women responded to the treatment with increased Hb values.

**Table 1:** Demographic information of women in the study

Age (years)	28.3 ± 7.3
BMI	28.3 ± 8.2
Gravidity	4 ± 3
Parity	2 ± 2
Mode of delivery	
Vaginal	38 (58.4%)
Elective Caesarean	19 (29.2%)
Emergency Caesarean	4 (6.2%)
Instrumental	4 (6.2%)
Oral iron supplements	31 (48%)
Iron intolerance	10 (15%)
Blood loss (ml)	413 ± 340
Gestational age at intervention (weeks)	34.3 ± 3.6
Haemoglobin at booking (12 weeks)	113.4 g/L
Ferritin at booking (12 weeks)	17 μg/L

Data are presented as n (%) or mean (± standard deviation).

Of the 65 women entered into the study, 18 (27.7%) women were defined as having severe anaemia (Hb <90 g/dl), while 12 women (18.5%) were defined as having moderate anaemia (90–94 g/dl) and the remaining 35 (55.8%) women had mild anaemia (95–116 g/dl)

**Figure 1:**
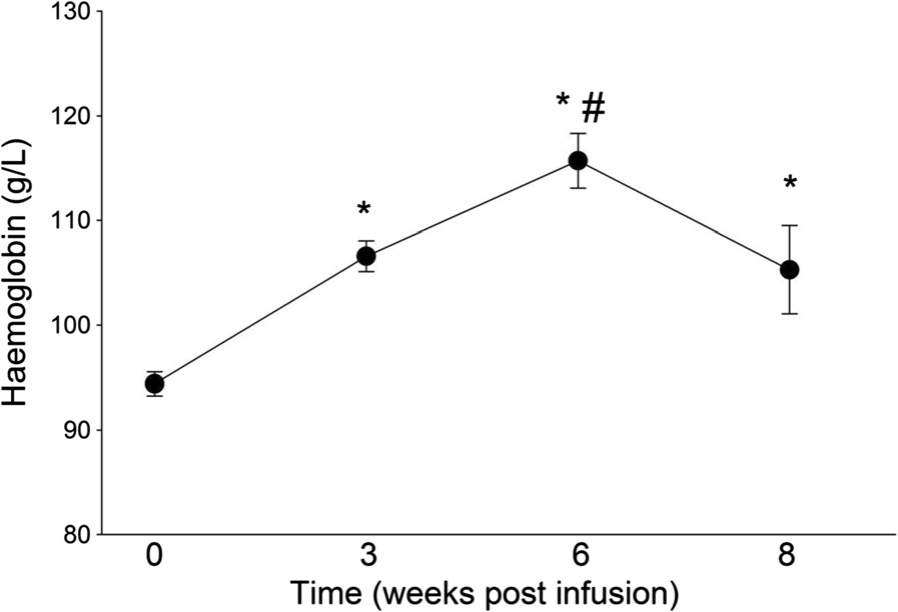
**Mean ± SEM haemoglobin levels (g/L).** *p < 0.01 compared to baseline (pre-infusion), #p < 0.01 compared to 3 weeks.

Changes in haemoglobin concentration over the post-infusion period are presented in Figure 1. The pre-infusion haemoglobin level was significantly lower than haemoglobin values measured at all subsequent visits (p < 0.01 in each case). There was a significant increase in haemoglobin levels from 3 to 6 weeks post-infusion (average increase 12 g/dl; p < 0.01). By 8 weeks post-infusion, these values had returned back to levels comparable with those observed at 3 weeks post-infusion, which were still significantly higher than pre-infusion levels. When IDA severity was included in the analysis, a similar pattern of results emerged (Table 2). For all three severity groups, haemoglobin levels increased post infusion at 3 and 6 weeks, to be significantly higher than baseline levels (p < 0.01 in all cases). However, the post-partum haemoglobin levels were only significantly higher than baseline in the women with mild IDA (p < 0.01), while haemoglobin levels in the moderate and severe groups had reverted back to pre-infusion levels. The analysis of the post-partum haemoglobin levels, however, was limited by small numbers in each group at this time point (mild n = 11, moderate n = 3, severe n = 4).

**Table 2:** Haemoglobin levels (g/L) across the testing period for women in the study, split by severity of iron deficiency anaemia at study enrolment

	**Gestational age at entry**	**Pre-infusion**	**3 weeks post infusion**	**6 weeks post infusion**	**8 weeks post infusion (post-partum)**
**Mild ≥95 g/L**	34 (4)	102.1 (1.0) n = 31	108.3 (3.9)* n = 28	120.6 (2.9)* n = 18	113.1 (4.2)* n = 11
**Moderate 90-94 g/L**	36 (2)	92.6 (0.4) n = 14	105.8 (3.0)* n = 13	108.4 (3.8)* n = 5	92.7 (12.4) n = 3
**Severe <90 g/L**	34 (3)	83.7 (0.9) 20	100.2 (3.3)* n = 17	110.0 (8.1)* n = 5	93.3 (8.1) n = 4

Data are presented as means (SEM). *p < 0.01 compared to pre-infusion haemoglobin levels.

Ferritin values increased significantly after the infusion (Table 3). As there was no strict protocol, post-partum ferritin levels were available for only 2 patients. However, despite the limitation, these values indicate reasonably replenished iron stores, with mean (±SD) levels of 151 μg/L (± 4.2).

**Table 3:** Ferritin levels (μg/L) across the testing period for women in the study

	**Booking**	**Pre infusion**	**Post infusion**
**Ferritinμg/L**	13.5 (13) n = 47	6.5 (3.9) n = 25	194 (316)* n = 24

Data are presented as means (SD). *p < 0.05 compared to pre-infusion levels.

All adverse reactions are presented in Table 4. No serious adverse effects were recorded in any of the 65 women receiving an infusion. Minor side effects occurred in 13 (20%) patients. One patient required medication with Metoclopramide for nausea and vomiting. All other adverse events were self-limiting. Fetal heart rate monitoring did not indicate a drug related adverse effect on the fetal heart pattern. Red blood cell transfusions were required by 3 women (4.6%) in the study cohort, all of whom had a significant peri-partum haemorrhage.

**Table 4:** Number of women experiencing a drug related adverse events following infusion with ferric carboxymaltose (total number of women infused n = 65)

**Adverse event**	**n (%)**
Any adverse event	13 (20)
Local (injection site irritation)	
Slight burning sensation	5 (8)
Systemic	
Hypotension	1 (1.5)
Headache	4 (6)
Nausea/Vomiting	1 (1.5)
Pruritus	2 (3)

Follow-up interview by telephone was conducted on 29 (44.6%) women in the post-partum period. Of these women, 19 (65.5%) reported an improvement in their wellbeing (48.3% reported feeling “much better”, 17.2% reported “a little better”, and 9 (31%) reported feeling “no different”) after the infusion. One woman was unwilling to provide information. None of the women reported feeling worse.

## Discussion

This is the first prospective study reporting on ferric carboxymaltose infusions in pregnancy. The key finding of our study is that in women presenting with IDA relatively late in pregnancy, a ferric carboxymaltose infusion prior to delivery significantly increased haemoglobin levels and improved iron stores. Further, we demonstrate that ferric carboxymaltose appears to be a safe and effective treatment modality for the correction of IDA, as no serious adverse events and only few minor adverse events reported. Reassuringly, patient satisfaction rating and improvement in perceived wellbeing assessed in the postnatal period was high

Many women develop iron deficiency during pregnancy, a condition that can have serious maternal and fetal implications [14]. In our cohort, first trimester booking bloods showed only discrete anaemia with mean Hb of 113.4 g/L, but all women studied developed moderate to severe IDA. The low mean ferritin at booking of 17 μg/L represent profound iron deficiency and reiterates the importance of ferritin as a screening tool. This finding should generally result in the initiation of iron supplementation. For some women oral iron supplementation appears to be sufficient to maintain adequate iron stores. However many women develop moderate to severe IDA despite oral iron supplementation (as demonstrated in the current study where 48% of women were on oral iron), or due to drug intolerance (15% in the current study), non-adherence or pre-disposing pathology such as malabsorption or inflammatory bowel disorders. For those women intravenous iron administration may be a more effective treatment modality.

To date no prospective, controlled clinical study has been performed using ferric carboxymaltose in pregnant women. A recent Cochrane review concluded that large, good quality trials, assessing clinical outcomes (including adverse effects) as well as the effects of treatment by severity of anaemia are required [18]. In the absence of these studies, observational safety and efficacy data may help identify potential benefits and risks. Two recent retrospective observational studies comparing ferric carboxymaltose to different intravenous iron preparations highlighted the safety and efficacy of ferric carboxymaltose [19,20].

The rapid delivery option of a large single dose of ferric carboxymaltose offers a promising treatment modality for pregnant women who need correction of iron deficiency and anaemia, over other IV iron formulations that have low dosage limits, such as iron sucrose (200 mg). The properties of ferric carboxymaltose may also reduce the burden on the patient and the health care system.

In obstetrics, red blood cell transfusions currently account for 3–4% of all transfusion events and the majority of these occur following post-partum haemorrhage (PPH) [21]. PPH is the leading cause of maternal mortality in obstetrics, and is estimated to occur at a rate of 13.1% [9]. Despite its enormous clinical utility, RBC transfusion is a treatment with well described adverse events and risk, and should ideally be avoided. Additionally blood is both costly and in ever increasingly short supply [22-24]. In the present cohort, only three patients (4.6%) required a RBC transfusion following a significant PPH. A recent large retrospective study revealed much higher transfusion rates of 7.5% in women with clinical PPH [25]. The current data suggests that improving Hb, even at a late stage of the third trimester may have shielded some mothers in our cohort from the risks of an allogeneic transfusion. This does not only spare resources, but also optimizes the health of women throughout and beyond her pregnancy into the challenging post-partum period [4,26].

## Conclusion

The data from this prospective case series is consistent with existing retrospective data that ferric carboxymaltose administration in the second and third trimester of pregnancy is likely to be safe and effective. In our study ferric carboxymaltose successfully corrected IDA prior to delivery. The intervention prevented significant post-partum anaemia in all women resulting in post-partum haemoglobin values higher than their pre-treatment antenatal values. Despite moderate to severe anaemia at presentation, labour associated blood loss was tolerated well resulting in low peri-partum RBC transfusion rates. No serious adverse events were recorded. Well-being also improved for the majority of women after the infusion.

## Abbreviations

IDA: Iron deficiency anaemia; Hb: Haemoglobin; RBC: Red blood cell; PPH: Post-partum haemorrhage.

## Competing interests

BF has received lecture honoraria or travel support in the last 5 years from New South Wales Department of Health, South Australia Department of Health, Australian Red Cross Blood Service, Australian National Blood Authority, Vifor Pharma Ltd., Glattbrugg, Switzerland, Fresenius Kabi GmbH, Bad Homburg, Germany.

No support was received from any organization for the submitted work; No other relationships or activities that could appear to have influenced the submitted work. JC, NH and GD have no conflict-of-interest to declare.

## Authors’ contributions

BF participated in the design, acquisition of data, communicated with participants and drafted the manuscript. JC participated in the design, acquisition of data and revising the manuscript critically for important intellectual content. NH performed the statistical analysis and helped to draft the manuscript. GD conceived of the study, and participated in its design and coordination. All authors read and approved the final manuscript.

## Pre-publication history

The pre-publication history for this paper can be accessed here:

http://www.biomedcentral.com/1471-2393/14/115/prepub
